# How to combat stigma surrounding mental health disorders: a scoping review of the experiences of different stakeholders

**DOI:** 10.1186/s12888-024-06220-1

**Published:** 2024-11-08

**Authors:** Alireza Hajizadeh, Homayoun Amini, Mahdiyeh Heydari, Fatemeh Rajabi

**Affiliations:** 1 Health Information Management Research Center, Tehran University of Medical Sciences, Tehran, Iran; 2 Department of Psychiatry, School of Medicine, Roozbeh Hospital, Tehran University of Medical Sciences, Tehran, Iran; 3 Social Determinants of Health Research Center, Clinical Research Institute, Urmia University of Medical Sciences, Urmia, Iran; 4 Department of Health Economics and Management, School of Public Health, Urmia University of Medical Sciences, Urmia, Iran; 5 Community Based Participatory Research Center, Tehran University of Medical Sciences, Tehran, Iran

**Keywords:** Mental health, Mental health disorders, Stigma, Discrimination, Intervention

## Abstract

**Background:**

The stigma associated with mental health disorders (MHDs) results in delayed help-seeking, limited access to health services, suboptimal treatment, poor treatment outcomes, and an increased risk of human rights violations. This scoping review summarizes qualitative research on the lived experiences of different stakeholders regarding strategies and interventions to combat stigma for people with MHDs.

**Methods:**

This study was a six-step scoping review using the Arksey and O’Malley framework. A comprehensive search of the following electronic databases was conducted to identify relevant records: PubMed, Scopus, Web of Science (WoS) and Google Scholar, as well as a manual search of the reference lists. All steps, including screening of eligible studies, data extraction, and analysis, were performed independently by multiple reviewers, with disagreements resolved by discussion. The data were synthesized based on the for-content synthesis guidelines.

**Results:**

A total of 25 studies were included in this review of the 32,976 initial identified citations. The included studies were from all countries (low, middle, and high income), stigmatized disorders (e.g., schizophrenia, bipolar disorder, etc.) and target populations (e.g., people with MHDs and their families, health care providers, the general community, and students and school members). The thematic synthesis revealed six types of interventions and strategies and 17 themes related to reducing stigma in patients on MHDs. Strategies and interventions were classified by patient (self-stigma), family (family stigma), healthcare professionals’ stigma, workplace stigma, public/societal stigma, and structural type of stigma (institutional stigma).

**Conclusions:**

This review contributes new evidence that should be considered in future interventions and policies to reduce stigma against MHDs. Multilevel and multistakeholder strategies and interventions are needed to reduce the stigmatization of MHDs.

**Supplementary Information:**

The online version contains supplementary material available at 10.1186/s12888-024-06220-1.

## Background

Currently, mental health disorders (MHDs) are increasingly being diagnosed as the leading cause of disease burden worldwide and disproportionately affect many people, especially poor people [1]. The Lancet Commission on Global Mental Health and Sustainable Development identified mental health as a fundamental human right and necessary for the growth of all countries. Therefore, this commission emphasized more investment in mental health aspects as part of universal health coverage (UHC) and the integration of these services into the global encounter with other health problems [2]. Based on conservative estimates, MHDs accounted for 654.8 million estimated cases in 1990 and 970.1 million cases in 2019, corresponding to an increase of 48.1% between 1990 and 2019 [3]. The COVID-19 crisis has also led to an increase in MHDs in the global population, especially among healthcare providers, patients with noncontagious chronic diseases, people in quarantine, COVID-19 patients and their families [4].

The leading contributors to MHDs worldwide are depressive disorders, anxiety disorders, bipolar disorder, schizophrenia and other psychotic disorders, dementia, attention deficit hyperactivity disorder and developmental disorders, including autism spectrum disorder [5]. MHDs can significantly impair people’s quality of life and can also lead to suicidal ideation, maladaptive behaviors, and burnout [6]. If MHDs are associated with stigma, there will be a reduction in the utilization of health services, a delay in seeking support, weak outcomes, suboptimal treatment, and an increased risk of individuals’ human rights violations in these patients [1].

Stigma is a negative form of labeling people or a group of people who excludes them from society based on physical or psychological differences or perceived differences [7, 8]. Additionally, stigma is defined as the connection among stereotypes, negative attitudes and discrimination against people living with MHDs in the community [9]. Stigma often places individuals in a stereotypical group, and the discrimination that individuals experience makes it difficult for them to demand any psychological intervention [10].

A review of the available literature shows that the prevalence of stigma against patients with mental disorders has been variable in different societies and cultures. The results of a systematic review and meta-analysis conducted in 2023 showed that pooled prevalence of internalized stigma was 29.05% in African countries, which in the subgroup analysis by country, Ethiopia had the highest prevalence of internalized stigma at 31.8%, followed by Egypt at 31.26%, and Nigeria at 24.31% [11]. In a study at Ethiopia (2022), 35.2% of patients with mental disorders reported experiencing high levels of perceived stigma [12]. Also, research results in Nepal (2019) showed that the overall prevalence of self-stigma in MHDs is 54.44%, which among those who had self-stigma 48% had mild self-stigma, 34.7% had moderate self-stigma and 17.3% had severe self-stigma [13]. Also, the results of another review (2021) showed that about one-third (31.3%) of people with a variety of mental disorders reported self-diagnosis, which was more common in Southeast Asia and the Middle East, and more common in Europe, Africa and North America for schizophrenia [14].

Due to the existence of a wide range of countries and cultural settings, the importance of understanding cultural differences has been emphasized to combat stigma and promote mental health awareness globally [15]. In many Asian societies, mental health issues are often seen as a sign of personal weakness or lack of self-control, with the stigma associated with mental illness being seen as a shame on the family [16]. In some African cultures, mental illness is often attributed to spiritual or supernatural causes such as curses or possession by evil spirits, which discourages patients from seeking psychiatric help [17]. In Arab societies, these diseases are seen as a form of divine punishment, and religious belief that perpetuates the stigma of mental health can lead to delays or avoidance of treatment as individuals may resort to religious or spiritual interventions [18]. Also, personal weakness or lack of will has been identified as one of the main perceived causes of mental illness in some Latin American cultures, which causes stigma and people refuse to receive medical care [19]. In Western culture, stigma stems mostly from misconceptions about MHDs, including the belief that people with MHDs are dangerous or unpredictable [20].

However, is now the time for action to be taken at multiple levels in all countries to reduce stigma. In this regard, there are various long-running MHD anti-stigma campaigns in high-income countries, such as ‘Time to Change’ in the UK, ‘Opening Minds’ in Canada, and ‘Beyond Blue’ in Australia, that have shown significant positive changes [21]. Like in developed countries, in low- and middle-income countries (LMICs), there are interventions targeting MHD stigma that need to increase efforts in these programs [22, 23]. There are many initiatives and plans that attempt to address the stigma of MHDs, such as improving mental health care and accessing and reducing stigma through education, advocacy, and research for people with mental illness [24]. Due to the magnitude of the problems resulting from MHDs, concerted activity on stigma is required to fund methodologically strong research that provides evidence to support decisions about investing in interventions to reduce stigma [25].

Similar study by Gyamfi et al. (2024), our initial survey of the available literature gave us indications that there was a paucity of empirical research evidence on the combat with stigma surrounding MHDs [26]. Also, few studies focusing on the perspective of people living with MHDs, relatives or mental healthcare providers, and other stockholders have been published [27]. Among the available evidence, our research is global and aggregates and synthesizes evidence on the lived experiences of various stakeholders at any type of stigma, so it can help patients and their families, societies, healthcare professionals and policymakers to combat stigma surrounding mental health disorders.

Considering the increase in the burden of MHDs and the importance of paying attention to lived experiences combating stigma, this scoping review aimed to synthesize the results of qualitative research on strategies and interventions that various stakeholders were taking into account to reduce stigma toward people living with MHDs. The present scoping review did not choose a pre-defined theoretical framework because combating stigma surrounding mental health disorders involves various theories and approaches tailored to different stakeholders, including individuals with mental disorders, their families, healthcare providers, policymakers, and the general public. Each theory has advantages and disadvantages and it may be more relevant to a specific group of stakeholders and to a specific kind of stigma. Therefore, this scoping review was exploratory in nature and aimed to map the existing literature on the lived experiences of various stakeholders, identify the key concepts, and highlight the gaps in the current research.

This study is part of a larger research project that aims to design a package of delivery strategies for evidence-based interventions to reduce stigma and discrimination against individuals living with priority MHDs in Iran.

## Criteria for considering studies for concept in this scoping review

We consider six types of stigma, namely, self-stigma, family stigma, healthcare professionals’ stigma, workplace stigma, societal stigma (public/social stigma) and structural or institutional stigma, and all analyses and sections of the study are based on these [1, 28–30]. The basis for the classification of mental illness in this review was the International Classification of Diseases (ICD-11), the 11th edition of a global categorization system for physical and mental illness published by the World Health Organization (WHO) [31]. The WHO defined stigma as “a sign of shame, disgrace or disapproval that results in an individual being rejected, discriminated against and excluded from participation in a number of different areas of society” [32]. We also used the definition of the WHO for intervention: “An act performed for, with or on behalf of a person or population whose purpose is to assess, improve, maintain, promote, or modify health, functioning or health conditions” [33]. In the current review, we distinguished between two concepts of intervention and strategy (coping style) to clarify the results. “Intervention” here refers to a defined and intentional action for reducing the negative consequences of stigma and is usually planned from outside of the patients, but “strategy” refers to conscious coping styles used to address and overcome struggles and difficulties in life and is not planned from outside of the patients. Therefore, we use the word “strategy” to address stigma at the patient or family level and the word “intervention” to combat stigma outside of patients.

## Materials and methods

### Study design

This study was a scoping review that was conducted based on the Arksey and O’Malley framework. To our best knowledge, there has been no extensive scoping review of strategies and interventions needed to address stigma in MHDs. Therefore, our aim in choosing Arksey and O’Malley framework was to cover all interventions or wide range of strategies at different levels to provide greater conceptual clarity about a specific topic or field of evidence. So, we summarized the qualitative results to cover the knowledge gap and provide the context for more specific systematic reviews and meta-analyses in futures. Arksey and O’Malley framework provide an excellent methodological foundation, and help to maximize the usefulness of our findings within healthcare research and practice. This framework includes six steps used for this scoping review: (1) clarification of the question of study; (2) identification of the relevant studies; (3) selection of the studies; (4) data charting; (5) collating, summarizing, and reporting; and (6) providing guidance and practical advice.

### Step 1: Clarification of the research question

To address this issue, the following question was asked when examining the relevant current evidence: What strategies and interventions have been suggested by mental health stakeholders with regard to their lived experiences to reduce stigma toward people living with MHD based on evidence from qualitative studies?

### Step 2: Identification of the relevant studies

We searched several electronic databases, including the Medline (PubMed), WoS and Scopus databases. We checked the references of the included studies to identify additional studies not retrieved by the preliminary searches (Reference by reference). In addition, Google Scholar, gray literature and other available information sources were also searched. The search in each database was conducted on titles and abstracts. To combine terms, Boolean operators (AND, OR and NOT) are used. The search terms and strategies for the databases are presented in Table 1 of the attached file.

**Table 1:** Inclusion and exclusion criteria

Criteria	Inclusion criteria	Exclusion criteria
Language	Only English language	-
Time period	From January 1, 2000, until the January 1, 2024	-
Study design	Qualitative research (Any data collection method (such as interviews, focus groups, diaries or online data collection) and any method of qualitative analysis of primary data (such as grounded theory, ethnography, thematic analysis, interpretive phenomenological analysis (IPA), framework approach, or narrative analysis).)	Review article, quantitative study, opinion, editorials, conference abstracts and letters articles
Domain specified	Providing information about interventions and delivery strategies for reducing the stigma and discrimination against individuals living with mental disorders	Studies focused on neurological and substance use disorders
Studies participants	Any participants (MHDs, patients’ family, healthcare providers, nonhealthcare providers, policymakers, managers, etc.) Discussing current strategies or recalling details retrospectively No age restrictions No restrictions on other physical or mental health comorbidities	-
Characteristics of studies	Studies that have a clear design and results	Studies/records whose full texts are not available
Setting and geography	No restriction to the setting and no restriction on geography	-
Place of published	Peer-reviewed scientific journals	-

The details on the inclusion and exclusion criteria are shown in Table 1. There are several reasons for focusing on qualitative evidence in this review. In this review, only qualitative data will be presented to provide a deeper analysis and understanding of the experiences of stigma among those living with mental disorders in societies and their families as well as other mental health stakeholders. A rigorous qualitative review can also uncover new understandings, often helping to illuminate ‘why’ and help build theory. Such a review can answer the question in the present review, ‘What are the interventions or strategies for reducing stigma based on the lived experiences of various mental health stakeholders?’ In the present research, we needed deep insight into interventions and strategies that could not be achieved through statistical studies, and we needed to focus on textual findings for the next stages of the project, which is to design a package of delivery strategies for evidence-based interventions to reduce stigma.

### Step 3: Study selection/screening

**Fig. 1:**
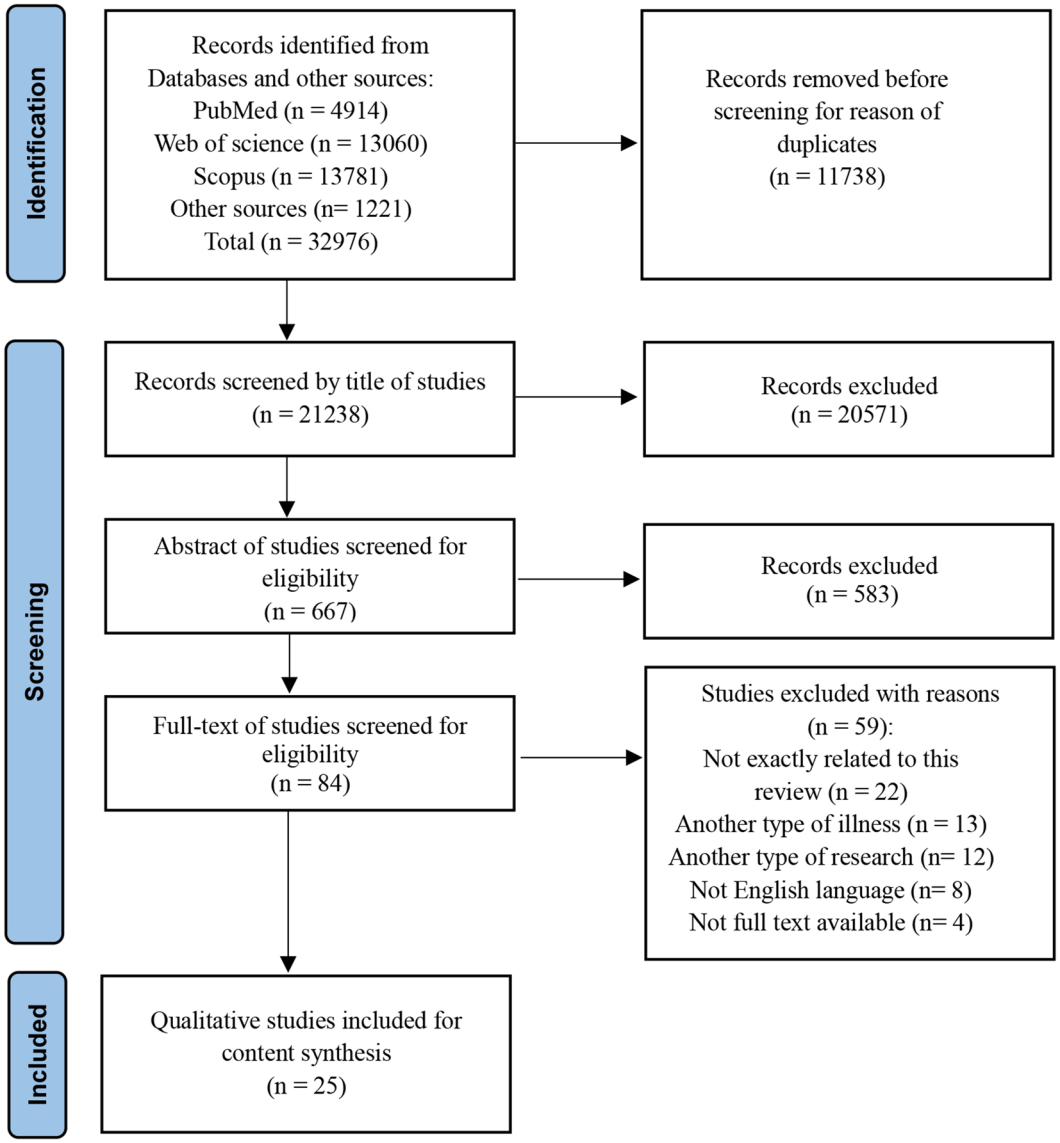
PRISMA diagram of the study search and selection process

All identified evidence was collated and uploaded to EndNote online software (version 8) to manage the screening process. Two authors (F.R., A.H.) independently screened the titles, abstracts, and full texts of all studies. When there was any discrepancy, the article remained on the list for review by a third author (M.H.). For any excluded study, at least one reason for exclusion was recorded. The search results, study selection, and inclusion process are reported in the PRISMA flow diagram for the scoping review process (Fig. 1).

### Step 4: Charting of the data

To extract data from qualitative studies, data extraction forms were manually designed using Microsoft Word 2010 software. Information extractable using the included study form: Author, year of publication, country of study, study design, participants, method of analysis and the results (Strategies and interventions applied or proposed).

### Step 5: Collating, summarizing, and reporting the results

The extracted data from the included studies were manually analyzed, summarized, and reported using the content analysis method. Content analysis is a method for identifying, analyzing, and reporting patterns (themes) within a text and is widely used for data analysis (27, 28). The steps for analyzing and coding the data included the following steps: familiarity with the text (immersion in study results), identifying primary themes, placing studies in determined themes, reviewing the studies of each area to complete the findings, and ensuring the reliability of the themes and the results extracted in each theme. Disagreements between the two authors were resolved through discussion, and if an agreement was not reached, the disagreement was resolved by a third author.

### Step 6: Providing guidance and practical advice

After completing the previous steps, based on the extracted results and opinions of the research team members, guidance was presented in the form of discussion, conclusion and suggestions.

## Results

### Systematic review

From the initial search results of 32,976 records, 25 qualitative studies were included in this review. Additionally, the full texts of 84 articles were examined, of which 25 studies were selected for the final analysis according to the inclusion and exclusion criteria. The details of the literature search and screening processes are presented in Fig. 1.

### Characteristics of the included studies

The included studies were published between 2002 and 2023. Most of these interventions were applied in high-income countries; however, the regions included the USA (*n* = 4), Iran (*n* = 3), Ghana (*n* = 3), Singapore (*n=2*), Germany (*n* = 1), New Zealand (*n* = 1), Zambia (*n* = 1), Turkey (*n* = 1), Canada (*n* = 1), Bellerose (*n* = 1), Brazil (*n* = 1), England (*n* = 1), Mexico (*n* = 1), Indonesia (*n* = 1), Spain (*n* = 1) and Nigeria (*n* = 1). Additionally, a joint study was conducted in one low-income country (Nepal), two lower-middle-income countries (India, Tunisia), one upper-middle-income country (Lebanon), and three high-income countries (Czech Republic, Hungary and Italy). In all the included studies, the phenomena of interest were the perception and actual experiences of stigma among people with MH-related disorders.

Participants in the included studies were recruited from all groups of stakeholders, including both inpatient and outpatient psychiatric professionals; people suffering from any type of MHD and their families; and students, teachers and healthcare providers, with the majority being recruited from people living with MHDs. Although two mixed methods studies were included, only qualitative findings and illustrations derived from individuals with a diagnosis of MHD were included in the review. All but one study applied interviews; the other used focus group discussions for gathering data. The most common analytic approaches were thematic (*n* = 12), alongside framework (*n* = 2), content (*n* = 8), grounded theory (*n* = 2), and narrative (*n* = 1) methods. Table 2 in the attachments summarizes the characteristics of the included studies.

**Table 2:** Themes and subthemes of interventions to reduce stigma toward people living with MHDs

Types of strategies/interventions	Theme	Subtheme
Self-stigma or perceived stigma	Individual strategies	• There’s nothing to be ashamed of • I’m not alone • Keep it private • Self-esteem of the patients has to be increased • Stigmatization fear has to be overcome • Doing introspective work • Taking full responsibility • Mental health individuals empowered by sharing Acceptance • Self-motivation • Standing up for themselves • Improve themselves and live life as per normal • Mental fortitude • Self-management • Religious practice • Resignation about illness • Self-concealment of the diagnosis (“live behind closed doors”) • Personal development
	Communication strategies	• Use existing family/peer support • Health care professionals sharing their experiences is beneficial • Avoidance of marriage • Sharing/encouraging disclosure to others • Nondisclosure of condition • Peer support networks enhancing resilience Seek social support • Avoidance of the rest of the family • Social distancing • Defending rights • Normalizing (Being natural) • Find a new support network • Tell certain people certain things • Aggression and reaction
	Awareness-related strategies	• Personal experiences foster mental health understanding Individual efforts in raising awareness • Control the flow of information • Media promoting mental health awareness.
	Delivery health services	• Medical care adherence • Mental health care–seeking action (Professional help) • Improving self-care • Request for assistance • Put health above what people think • I’m proud that I’m getting help
Family stigma	Having supportive behaviors	• The diagnosis should be accepted by family members • Supportive spouses and family • Giving successful examples • Meeting/coming close to patients • Respecting to patients • Working on recovery • Prayer’s practice • Assistance in the life of people living with MHDs • Provision of better access to appropriate information • Psychoeducation by creative techniques such as movies or videos
	Communication strategies	• It should be disclosed • Empathetic communication • Peer interaction and support • Reframing words in interactions
Healthcare professionals Stigma	Training and education of health service providers	• Various professionals should be given education • Psychiatrists has to be informative • There must be in-service trainings • Changing the attitudes of health care providers • Need for regular and continuous training and supervision for PCPs on topics such as psychiatric medication • early and mandatory training on stigma in MHDs in the medical school curriculum Courses should be taught each year to reinforce learning • Psychiatrists should receive psychotherapy as attitudes could be related to their own personality traits
	Providing appropriate health services	• Changes in mental health care • Providing of screening • Integration of psychiatric wards in the general hospitals • Promoting supportive services • Consultation-liaison has to be put into practice • A more general diagnosis should be written on the prescriptions • Providing suitable environmental support such as having a separate room for counseling to ensure privacy • Use community-centered care • Use in-home services • Providing psychosocial support to someone in need • Communicating with service users, and addressing stigma and self-care • Talking to a psychologist was perceived as beneficial in empowering parents
	Promoting empathy and collaboration	• Build trust and rapport • Using patient-centered communication • Listening/caring • Paying attention to language of patients • Empathy in interactions with service users • Delimiting the disciplines and preventing the involvement of nonexperts • Increased collaboration of PCPs with mental health specialists and their supervision • Rationalization • Tactical or planned ignoring • Tailoring the discussion of mental health to patients’ preferred understanding • Psychiatrists should be resistive Continuous evaluation of PCPs works as essential as the training • Involving patients, or their personal testimonies, to increase trainees’ awareness of their attitudes
Workplace stigma	Empowering employees in the workplace	• Employers, supervisors or managers have adequate knowledge of MHDs • Improve access of employers to education in managing employees with MHDs • Discussions about the purpose and use of extrinsic support are held between supervisors and employees • Issues around consent are fully explored • Use of extrinsic supports for the purposes of counteracting the effects of internalized stigma • Education is provided that progressively challenges the employee’s own attitudes and prejudices • Policies that encourage employers to hire and support of patients with MHDs
	Providing suitable workplaces setting	• Removal of Declaration of MHDs in Job Application and Scholarship Forms • The workplace culture promotes open discussion around staff support and accommodation of support needs • Opportunities are created to foster the development of positive relationships within the workplace • There is mentoring from other people who have MHDs and who successfully maintain employment • Opportunities are created where employees feel safe to explore support strategies
Public/societal stigma	Community engagement and education	• Education, training and teaching of community • Prevention of stigma by raising mental health awareness • Use educational campaigns • Promotion of knowledge regarding mental disorders within the society • Media has to be made aware, it should not encourage the stigmatization • Emphasis on changing attitudes and literacy of public • Public education campaigns through media such as television and radio • Social contact-based education
	Practical actions in society	• Normalizing discussion of mental health • Introducing recovered patients • Fostering a supportive social environment • Spiritual well-being • Working on social inclusion • Changing the culture • Opportunities to interact and social contact • Creating a common language • Explanation of nature of MHDs to society • Open communication • Support for the ill and their relatives • Testimonies of success stories by people with MHDs
	Role of key actors/determinants	• Use of social media • Implementation of public campaigns • The role of books and educational materials • The role of popular individuals • Celebrity endorsement • Role of ministry of health, municipality and other organizations
Institutional stigma	Supportive policymaking	• Transformation of mental health policy and legislation • Advocating for public health policies • The legislation of anti-discriminatory laws • Integrated reform of structures and policies • Evidence-based policies
	Playing a positive role by stakeholder organizations	• Advocacy by influential figures or groups • Efforts should be leads by organizations other than psychiatric experts. • Associations and institutions must take an active role • Utilizing the potential of religious clergymen • Organizations that have larger influence should partner with mental health experts
	Operational measures	• The dissemination that MHDs are not transmissible and dangerous • The clarification that people with MHDs want to be treated with respect and equality • Having a support network (See peer support) • Expanding access to and improving the quality of mental health care • Control and supervision of mental health centers • Expanding the social and economic opportunities of the mentally ill • Education acting on an organizational level • Establishing cultural committees, launching campaigns, and determining a support ambassador • Holding festivals • Budget and insurance coverage • Necessity to devise appropriate tariffs for mental health services • Consideration of the social rights of patients • Establishing committee and secretariat • Actions based on research • Using successful projects as a pattern • Emphasis on having systematic and massive programs

### Results of content analysis

In this section, the identified strategies and interventions were then subjected to content synthesis to produce a comprehensive set of aggregated findings that could be applied as a basis for evidence-based practice. The analysis of the included studies revealed six types of strategies and interventions and 17 themes related to the question of this scoping review (Table 2).

### Theme 1: Strategies for self-stigma or perceived stigma

Self-stigma refers to negative attitudes, including internalized shame that people with mental illness have about their own condition. The analysis performed in the present review identified and categorized several strategies at the level of mental patients to control self-stigma. The main themes presented to reduce self-stigma are as follows: individual strategies, communication strategies, awareness-related strategies, and delivery health services.

### Theme 2: Strategies for addressing family stigma

Family member support is a specific type of group support that focuses on the relationships among patients living with MHDs. The attitude of the family plays an important role in reducing or increasing the stigma and distress the person may experience and in helping the person come to terms with the health condition. Having supportive behaviors and interventions related to family interactions are the two main issues at this level to reduce the stigma toward mental patients in families.

### Theme 3: Interventions for professionals (The stigma of healthcare professionals)

This category illustrates the stigma from healthcare providers (in any setting of health centers, including primary health care, therapeutic, diagnostic, and specialized services) toward their patients with MHDs. To present a more detailed characterization of the types of interventions identified in this analysis, we categorized the interventions targeted at health care providers into three main themes: training and education of health service providers, providing appropriate health services, and promoting empathy and collaboration.

### Theme 4: Interventions for workplace stigma

One context of intervention to reduce stigma against patients with mental illness is the workplace, which implies recognizing that for its reduction, work must be done at the individual type (microsystem) as well as in the group and interactive context (mesosystem). Based on the analysis of the results, we categorized the main themes of this area in the form of empowering employees in the workplace and providing suitable workplace settings, which themselves have subthemes and can help reduce stigma in the work setting.

### Theme 5: Interventions for public/societal stigma

Public stigma refers to negative or discriminatory attitudes that others have about mental illness in society. The findings of this review have important implications for reducing the stigma associated with experiencing mental health conditions in society. We classified interventions at this level into three main themes: community engagement and education, practical actions in society, and the role of key actors (determinants) in reducing stigma.

### Theme 6: Interventions for institutional stigma

Institutional or structural stigma is more systemic and involves policies of the government and related organizations that intentionally or unintentionally limit opportunities for people with MHDs during their lives. Based on the results of the analysis, supportive policymaking, which plays a positive role by stakeholder organizations, and operational measures are the main structural interventions that can be used to reduce stigma toward mental patients.

## Discussion

This scoping review aimed to identify and synthesize the qualitative literature available regarding strategies and interventions to reduce stigma toward people living with MHDs. This manuscript is a part of the national mega project in Iran, which seeks to design a policy package to deal with stigma in MHDs through identifying delivery strategies for evidence-based interventions. We needed interventions and strategies that are in-depth and used in the design of the framework to send to policy makers. Therefore, we used the results of qualitative studies to synthesize the best evidence for tackling stigma in MHDs with a deeper understanding of international experiences.

By analyzing the qualitative studies, we sought to interpret, conceptualize and better understand strategies and interventions in concepts, thoughts, experiences of stockholders. We classified appropriate multilevel interventions targeting patients with mental health disorders, their family members, healthcare professionals, workplaces, the general public, and structural levels that might be applied to decrease the harmful influence of related stigma toward any type of mental health problem. All these interventions/strategies will be localized and presented in order to reduce stigma towards patients with MHDs.

The first group of strategies focused on patients on MHDs as a target group for the empowerment and reduction of self-stigma. Different strategies to cope with stigma were presented, including seeking support from accepting members of their existing social networks, not feeling alone or ashamed, and viewing their own health as more important than the reactions of others [34]. At the personal level of stigma, providing balanced psychoeducation about mental illness to patients may help patients better address the consequences of stigma. However, psychoeducation needs to be balanced and contextualized, taking into account the biological, psychological, and social factors that influence mental health conditions [35]. One of the other interventions to reduce self-stigma is communicating with people, as it has been stated that sharing experiences of stigma and mental health problems with others can have valuable results [36].

Another source of stigma against mental disorder patients is their family or relatives, and interventions of this type are also presented to reduce stigma in this review. Family members can use flexible coping strategies depending on their personal resources, motivation, and willingness to disclose [37]. For example, schizophrenia is one of the most common severe mental illnesses, and family members play an essential role in helping individuals support the development of family organizations and the lives of people living with schizophrenia (PLS) [38]. To decrease the burden of stigma in the private lives of family members of people living with MHDs, evidence emphasizes psychoeducation for patients and their families through creative techniques, including movies or videos on mental health, to improve awareness and public education campaigns through mass media such as television and radio [39].

Stigma against people with mental health disorders in health centers can have negative effects on the health and care of these patients, which needs to be considered by health providers. Psychiatrists involved in the study in Turkey (2010) highlighted their informative and resistive role in anti-stigma interventions and suggested educational strategies for different groups in collaboration with various institutions [40]. Among health professionals, primary healthcare providers can also reduce the burden of stigma, which is emphasized by increased training of PCPs, regular and continuous training and supervision, and increased collaboration with and supervision by mental health specialists at this level [39]. The evidence is more clear for interventions regarding decreasing stigma since most of the included studies showed effective strategies such as the integration of psychiatric wards in general hospitals to prevent stigma against patients living with MHDs [41]. Also, it is vital to pay attention to the training of students in healthcare careers for use effectiveness interventions with mandatory professional training, with the active involvement of the teacher in charge and experts by experience, can be a valuable way to promote humanized and non-stigmatizing treatment [42].

According to previous evidence, mental health stigma is also known in the workplace and can have a negative impact on absenteeism, presenteeism, and turnover, with a great economic cost to organizational structures [43]. In workplaces, employers and service providers should ensure that people with MHDs receive the necessary support and education to become fully assimilated members of organizations [44].

Moreover, our findings categorized interventions at the level of public society to promote methods for reducing stigma in MHDs. Increased mental health awareness in communities, social contact, and advocacy by influential figures or groups should be applied as stigma reduction interventions in different contexts [45]. Social beliefs and attitudes about people with mental illness are predominantly negative; in these regards, interventions such as changing culture, communication measures, social media use, education, and training are recommended [46–48]. Also, our finding illustrated that increasing mental health awareness, social contact, advocacy by influential figures or groups, and legislating anti-discrimination laws are international interventions that were used to disrupt the process of stigma in countries around the world [45]. The role of stakeholders is also prominent for adopting strategies to combat stigma towards people with MHDs, as the previous evidence has emphasized the role of non-governmental organizations [41, 49].

To address stigma attitudes among patients with MHDs, several domains of structural factors can be applied to structure policy discussions focused on paths to reduce stigma at the top level [50]. As a policy at the national level, since 2010, the Argentine government has approved and implemented the National Law on Mental Health with the intention of allowing individual freedom in deciding admission to psychiatric facilities [51]. LMICs have focused on the implementation of national anti-stigma strategies for enhancing mental health knowledge and attitudes toward individuals living with MHDs [52]. Additionally, high-income countries have used several programs to reduce stigma against MHDs, such as the successful Open Minds program in Canada [53]. It is essential to take into account some coping strategies used by patients who may be a sign of self-stigma, such as “avoidance”, and must be considered as the objectives for change by appropriate interventions. The findings of the present review have also emphasized supportive policymaking, providing more practical recommendations for policymakers and practitioners to use to frame strategies. For example, the evidence has explained that although the burden of mental disorders is much higher than physical diseases [54], there is systematic discrimination in the budget al.location among policy makers and it requires policy interventions [55, 56].

Stigma towards MHDs at different levels that was mentioned is affected by cultural norms, religious beliefs and social attitudes on its manifestations and consequences [57]. Mental health issues may be differently depending on countries’ cultural backgrounds, for example, in Asian cultures mental health issues are seen as a sign of personal weakness or lack of self-control [16]. Also, in African cultures, the causes of mental illnesses are spiritual or supernatural (e.g. possession by evil spirits) [17], which in Arab cultures is also considered a form of divine punishment [18]. Understanding and addressing cultural differences in perceptions of mental health is essential. It should be emphasized the need for tailored interventions that respect and incorporate cultural beliefs and practices, making it relevant for healthcare providers and community organizations. By acknowledging cultural variations in different countries, more culturally appropriate and effective strategies/interventions can be developed to combat stigma and improve mental health care [57].

### Limitations

There are some limitations in this scoping review that are relevant to restrictions in the existing evidence that qualitatively explores the stigma against MHDs. Therefore, the subjectivity of the findings must be considered when interpreting the results of this review. Actually, caution is needed in generalizing the results of qualitative studies for stakeholders, especially policy makers. Another limitation is that only studies in the English language were considered. Additionally, it was not possible for the authors to access the PsycINFO database, which may have resulted in the loss of evidence.

### Recommendations for future research

Research through qualitative methodologies is useful for understanding perceptions and experiences of interventions for addressing stigma in MHDs; however, few qualitative studies have been conducted in low-income or middle-income economies. The findings provide an in-depth overview of MHD stigma reduction interventions, providing researchers in diverse resource-poor settings with additional knowledge to aid in planning such interventions. Future studies should pay attention to the differences in society’s culture in the use of interventions and explore the creation of culturally sensitive anti-stigma interventions to survey which areas of these policies are most effective and easiest to implement. Additionally, evidence regarding the effectiveness of policies and the outcome of legislation by governance in mental health stigma reduction is still lacking.

Any type of research with different methods may also be necessary in diverse countries to appraise the implementability, acceptability, and effectiveness of educational programs or other interventions to decrease mental illness stigma. Mass media play a key role in the lives of societies, and more research is needed to explore the effects of mass media interventions on stigma and to better understand which types of mass media interventions are useful. The COVID-19 pandemic has had a negative effect on people’s mental health, and future research needs to be conducted in various settings, such as workplaces, homecare, health centers, schools and universities, and other societal settings.

## Conclusion

The themes emerging from the analysis acknowledge the variability within strategies and interventions to cope with stigma against people living with MHDs. It is expected that the results of this review will inform future research, and practice to address MH-related stigma in societies, as well as approaches for improving the delivery of responsive healthcare services and care for consumers with MHDs and their providers or families.

Additionally, the strategies and interventions suggested by different mental health stakeholders and categorized in this review have the potential to help patients living with MHDs seek health care and guide the design of policies so that they improve the overall patient experience; however, it is necessary to examine these strategies and interventions in well-designed experimental studies. It seems that interventions such as education, contact, public campaigns, raising awareness training and proper public policies have been more recommended and can have favorable outcomes for people with MHDs at all types of stigma. Stigma toward MHD patients appears from various sources, such as families, health care providers, workplaces, policymakers and the general society, and interventions should be used specifically in all these groups.

## Electronic Supplementary Material

Below is the link to the electronic supplementary material.

Supplementary Material 1

## Data Availability

Data is provided within the supplementary information files.

## Abbreviations

MHDs: Mental Health Disorders
WoS: Web of Science
UHC: Universal Health Coverage
COVID-19: Coronavirus Disease 2019
LMICs: Low- and Middle-income Countries
ICD: International Classification of Diseases
WHO: World Health Organization
PRISMA: Preferred Reporting Items for Systematic Reviews and Meta-Analysis
FGD: Focus Group Discussion
PCPs: Primary Care Providers
