# Supplementary material for: How to combat stigma surrounding mental health disorders: a scoping review of the experiences of different stakeholders
Source: BMC Psychiatry. 2024 Nov 8;24:782. doi: 10.1186/s12888-024-06220-1 (PMC11549754; doi:10.1186/s12888-024-06220-1)
Supplement: Supplementary file 1 — Supplementary Material 1 [file 12888_2024_6220_MOESM1_ESM.docx]

**Table 1. Search terms and strategy for the databases**

| **Database** | **SET** | **STRATEGY** | **RESULT** |
| --- | --- | --- | --- |
| **PubMed** | #1 | ((stigma*[title/abstract] OR discriminat*[title/abstract] OR prejudic*[title/abstract] OR stereotyp*[title/abstract] OR "social perception"[title/abstract] OR "social distance"[title/abstract] OR "social rejection"[title/abstract] OR "social isolation" [title/abstract])) | 398,106 |
|  | #2 | (("mental disorder"[title/abstract] OR "mental illness"[title/abstract] OR "mental sickness"[title/abstract] OR depression[title/abstract] OR "mood disorder"[title/abstract] OR anxiety[title/abstract] OR Schizophrenia[title/abstract] OR "psychotic disorder" [title/abstract] OR "bipolar disorder"[title/abstract]) OR "Obsessive-compulsive disorder"[title/abstract] OR Suicide [title/abstract])) | 811,031 |
|  | #3 | (intervention[Title/Abstract]) OR (strateg*[Title/Abstract]) | 2,288,053 |
|  | #4 | **#1 AND #2 AND #3** | 5,247 |
|  | #5 | **With filters from 2000 to now+ English language** | 4,914 |
| **Scopus** | #1 | ( TITLE-ABS-KEY ( stigma* ) OR TITLE-ABS KEY ( discriminat* ) OR TITLE-ABS-KEY ( prejudic* ) OR TITLE-ABS-KEY ( stereotyp* ) OR TITLE-ABS-KEY ( "social perception" ) OR TITLE-ABS-KEY ( "social distance" ) OR TITLE-ABS-KEY ( "social rejection" ) OR TITLE-ABS-KEY ( "social isolation" ) ) | 993,792 |
|  | #2 | ( TITLE-ABS-KEY ( "mental disorder" ) OR TITLE-ABS-KEY ( "mental illness" ) OR TITLE-ABS-KEY ( "mental sickness" ) OR TITLE-ABS-KEY ( depression ) OR TITLE-ABS-KEY ( "mood disorder" ) OR TITLE-ABS-KEY ( anxiety ) OR TITLE-ABS-KEY ( "psychotic disorder" ) OR TITLE-ABS-KEY ( "bipolar disorder" ) OR TITLE-ABS-KEY ( "obsessive-compulsive disorder" ) OR TITLE-ABS-KEY ( suicide ) ) | 1,580,769 |
|  | #3 | ( TITLE-ABS-KEY ( intervention ) OR TITLE-ABS KEY ( strateg* ) ) | 5,800,886 |
|  | *4 | #1 AND #2 AND #3 | 14,991 |
|  | *5 | **With filters from 2000 to now+ English language** | 13,781 |
| **Web of science** | #1 | (((((((TS=(stigma* )) OR TS=(discriminat*)) OR TS=(prejudice*)) OR TS=(stereotyp*)) OR TS=("social perception")) OR TS=("social distance")) OR TS=("social rejection")) OR TS=("social isolation") | 783,106 |
|  | #2 | (((((((((TS=("mental disorder")) OR TS=("mental illness")) OR TS=("mental sickness")) OR TS=(depression )) OR TS=("mood disorder")) OR TS=(anxiety )) OR TS=("psychotic disorder")) OR TS=("bipolar disorder")) OR TS=("obsessive-compulsive disorder")) OR TS=(suicide ) | 1,072,743 |
|  | #3 | (TS=(intervention)) OR TS=(strateg*) | 4,894,395 |
|  | #4 | #1 AND #2 AND #3  **With filters from 2000 to now+ English language** | 13,060 |
| **Another sources (e.g. google scholar, gray, manual search, reference by reference)** | | | 1221 |

**Table 2: Data extraction form of included studies**

| **Authors’ (Reference)** | **Year of publication** | **Country** | **Study design** | **Participants** | **Type of mental disorders** | **Results (Interventions and strategies applied or proposed)** |
| --- | --- | --- | --- | --- | --- | --- |
| Raingruber, B (1). | 2002 | USA | Qualitative study with narrative approach | 50 clients and providers | People with depression and/or suicidal ideation | - Open communication - Community awareness - Education, and prevention - Use community-centered care - Use in-home services |
| Angermeyer et al (2). | 2003 | Germany | FGD with thematic approach | 122 members of advocacy groups | Schizophrenia | - Communication measures - Support for the ill and their relatives - Changes in mental health care - Education and training - Control and supervision |
| Caltaux (3). | 2003 | New Zealand | Qualitative study with thematic approach | Employers and service providers | All type of mental disorders | - The workplace culture promotes open discussion around staff support and accommodation of support needs. - Opportunities are created where employees feel safe to explore support strategies. - Employers, supervisors or managers have adequate knowledge of and/or access to education in managing employees with mental illness. - Opportunities are created to foster the development of positive relationships within the workplace. - Discussions about the purpose and use of extrinsic support are held between supervisors and employees and issues around consent are fully explored. - Over time, the use of extrinsic supports (for the purposes of counteracting the effects of internalized stigma) fades, preferably to the point of being unnecessary. - Education is provided that progressively challenges the employee’s own attitudes and prejudices about mental illness. - There is mentoring from other people who have mental illness and who successfully maintain employment. - There are discussions about, and provision of, comparative data or facts regarding ‘ordinary’ employee entitlements. |
| Alvidrez et al (4). | 2008 | USA | Qualitative study with thematic approach | 34 public-sector Black mental health consumers | All type of mental disorders | Strategies to deal with stigma:   - Put health above what people think - There’s nothing to be ashamed of - I’m not alone - I’m proud that I’m getting help - Seek social support - Use existing family/peer support - Find a new support network - Control the flow of information - Tell certain people certain things - Keep it private - Be a role model/advocate for others |
| Kapungwe et all (5). | 2010 | Zambia | Qualitative study with grounded theory approach | 50 semi-structured interviews and 6 FGD with key stakeholders | All type of mental disorders | - Use education campaigns - Transformation of mental health policy and legislation - Expanding the social and economic opportunities of the mentally ill |
| Saillard (6) | 2010 | Turkey | Qualitative study with grounded theory approach | 9 psychiatrists and 8 assistant psychiatrists | All type of mental disorders | Strategies for the society (against the enacted stigma):   1. Education:  - Various professionals should be given education - Psychiatrists has to be informative - Media has to be made aware, it should not encourage the stigmatization - The nature of illness must be explained to society   Strategies for the patient and their relatives (against the perceived stigma)   - Associations must take an active role - The diagnosis should be accepted - It should be disclosed - Stigmatization fear has to be overcome - Self-esteem of the patients has to be increased   The improvement of health services (against the enacted stigma)   - Medical school curriculum has to be revised - Psychiatry internship has been prolonged - There must be in-service trainings - Psychiatrists should be resistive - Consultation-liaison has to be put into practice - A more general diagnosis should be written on the prescriptions |
| Corbière et all (7) | 2012 | Canada | Mixed method (Survey and interview) with thematic approach | 253 stockholders | All type of mental disorders | Theme: Education   - Educating/teaching - Giving successful examples - Acting on an organizational level - Paying attention to language   Theme: contact   - Sharing/encouraging disclosure - Meeting/coming close to   Theme: Protestation   - Defending rights - Reframing words   theme: Person centered   - Normalizing - Accepting/respecting - Listening/caring   Theme: Working on recovery and social inclusion   - Working on recovery - Working on social inclusion   Theme: Reflexive consciousness   - Doing introspective work - Being natural |
| Tawiah et al (8). | 2015 | Ghana | Mixed method with thematic approach | 277 mental health patients and caregivers and in-and mental health professionals | All type of mental disorders | - The most predominant strategy used by mental health patients was social (88%). This comprises of supportive spouses and family (23%), prayers (21%), aggression and reaction (14%), avoidance of marriage (13%) and others (16%). Economic strategies (i.e., fore go meals and animal rearing) form only 7% and psychological strategies (i.e., smoking marijuana and over sleeping) were 5%. |
| Krupchanka et all (9) | 2016 | Bellerose | Qualitative study with thematic approach | 20 relatives of people diagnosed with schizophrenia | People living with schizophrenia (PLS) | - Concealment (“live behind closed doors”) - Avoidance of the rest of the family - Taking full responsibility and sacrificing one’s personal life - provision of better access to appropriate information - Assistance in the life of people living with schizophrenia (PLS) |
| Taghva et al (10). | 2017 | Iran | Qualitative study with content approach | Experts of Mental Health, Social Health and Addiction (MEHSHAD) in the Ministry of Health and Medical Education (MoHME) | All type of mental disorders | Emphasis on education and changing attitudes   - Education and changing the attitudes of health care providers - Public education - Utilizing the potential of Islamic clergymen   Changing the culture   - Establishing cultural committees, launching campaigns, and determining a support ambassador - The role of media - The role of books and educational materials - Holding festivals - The role of popular individuals - Introducing recovered patients - Creating a common language   Promoting supportive services   - Budget and insurance coverage - Necessity to devise appropriate tariffs for mental health services - Consideration of the social rights of patients   Role of various organizations and institutions   - Ministry of Health and Medical Education - Municipality - Islamic Development Organization - Other organizations - Integrated reform of structures and policies - Establishing committee and secretariat - Delimiting the disciplines and preventing the involvement of non-experts   Integration of psychiatric wards in the general hospitals   - Emphasis on having systematic and massive programs   Evidence-based actions   - Research actions - Using successful projects as a pattern |
| Vedana et all (11) | 2017 | Brazil | Qualitative study with thematic approach | 46 Brazilian adults with mental disorders | All type of mental disorders | - Promotion of knowledge regarding mental disorders within the society - The dissemination that these disorders are not transmissible - The clarification that people with mental disorders are not dangerous and want to be treated with respect and equality. |
| Huggett et al (12). | 2018 | England | Qualitative study with thematic approach | Two focus groups of 13 people with experience of mental health problems and stigma | All type of mental disorders | ‘Taking back control’   - Sharing experiences of stigma and mental health problems were helpful - Talking to a psychologist was perceived as beneficial in empowering participants to continue moving forwards despite the impact of stigma - Gain direct personal experience and knowledge of mental health problems - Feel proud of their mental health problems and empowered to disclose - Disclosure of their mental health problems helped them cope with stigma and accept their condition   Having a support network (see Peer support)   - Peer support networks and their ability to help develop psychological resilience to overcome stigma |
| Bonsu, A. S.  And Salifu Yendork, J. (13). | 2019 | Ghana | Qualitative study with content approach | 10 mental health professionals and 10 family caregivers | All type of mental disorders | - Five different strategies (i.e. rationalization, tactical or planned ignoring, self-motivation, acceptance and the use of religion) were identified as coping strategies used to manage the unfavorable situations experienced by caregivers by virtue of their association with persons with mental illness |
| Farsi et all (14) | 2020 | Iran | Qualitative study with content approach | 14 Stakeholders who contact with psychiatric patients, a recovered patient, and a family member | All type of mental disorders | - Emphasis on education and changing attitudes - Changing the culture - Promoting supportive services - Role of various organizations and institutions - Integrated reform of structures and policies to improve the performance of custodians - Evidence-based actions |
| Ong et all (15) | 2020 | Singapore | Qualitative study with thematic approach | 42 People with mental illness (PMI) | All type of mental disorders | Individual strategies to reduce stigma (self-stigma)   - Non-disclosure of condition - Standing up for themselves - Individual efforts in raising awareness - Improve themselves and live life as per normal |
| Koschorke et all (16) | 2021 | One low-income country (Nepal), two lower-middle income countries (India, Tunisia), one uppermiddle- income country (Lebanon), and three high-income countries (Czech Republic, Hungary, Italy) | Qualitative study with framework approach | 248 participants: 64 primary care providers, 11 primary care facility managers, 111 people with mental illness, and 60 family members of people with mental illness. | All type of mental disorders | - More training of Primary care providers’(PCPs) in mental healthcare - Need for regular and continuous training and supervision for PCPs on topics such as psychiatric medication, providing psychosocial support to someone in need, communicating with service users, and addressing stigma and self-care. - More collaboration with and supervision by mental health specialists - Continuous evaluation of their work as essential as the training - providing suitable environmental support such as having a separate room for counselling to ensure privacy - Psychoeducation for service users and their families, using creative techniques such as movies or videos on mental health to raise awareness, and public education campaigns through media such as television and radio. - Improve PCPs effective communication and empathy in interactions with service users |
| Shahwan et all (17) | 2021 | Singapore | Qualitative study with thematic approach | 42 People with Lived Experience and Caregivers | All type of mental disorders | - Rising mental health awareness: “who and how” and “what” of this approach - social contact: celebrity disclosures-testimonies of success stories by people with mental health conditions-opportunities to interact with them - advocacy by influential figures or groups - Legislation of anti-discriminatory laws: Removal of Declaration of Mental Illness in Job Application and Scholarship Forms-Policies That Encourage Employers to Hire and Support PWLE in Workplaces |
| Commey et all (18) | 2022 | Ghana | Qualitative study with framework approach | 9 people with schizophrenia | People with schizophrenia | - Mental fortitude - Adherence (medical care) - Spiritual well-being |
| Lagunes-Cordoba et all (19) | 2022 | Mexico | Qualitative study with thematic approach | 29 psychiatric trainees | All type of mental disorders | - Inclusion of early and mandatory training related to mental health stigma in their curricula - Courses should be taught each year to reinforce learning - Involving patients, or their personal testimonies, to increase trainees’ awareness of their attitudes - Psychiatrists should receive psychotherapy as attitudes could be related to their own personality traits and, because there is also a need to take care of psychiatrists´ own mental health |
| Withers et all (20) | 2022 | USA | Qualitative study with content approach | 15 university students | All type of mental disorders | - Educational campaigns - Peer interaction and support - Counseling - Screening - Self-management |
| Aiyub et all (21) | 2023 | Indonesia | Qualitative study with content approach | 30 participants, including students, teachers, health professionals and lecturers | All type of mental disorders | - Increasing mental health literacy (MHL) - Reducing the harmful effects of stigma - Fostering a supportive social environment - Expanding access to and improving the quality of mental health care - Advocating for public health policies |
| Arboleya-Faedo et all (22) | 2023 | Spain | Qualitative study with content approach | 14 outpatients that suffer from chronic psychosis | All type of mental disorders | - Passive coping: Religious Practice-Resignation about illness - Active coping: Seeking professional help-Improving self-care - Avoidant coping: Social distancing- Self-concealment of the diagnosis - Ineffective coping: Attempting self-harm |
| Latifian et all (23). | 2023 | Iran | Qualitative study | Participants (n = 27), 4 were bipolar patients, 20 bipolar patient’s family members and 3 were mental health care professionals | Bipolar disorder patients | - Awareness - Request for assistance - Personal development - Treatment adherence - Responding to patient demand |
| Pederson et all (24) | 2023 | Nigeria | Qualitative study with content approach | Student volunteers from the university (n = 82) | All type of mental disorders | - Social media - Public campaigns - Normalization of mental illness - Social contact-based education |
| Phelan et all (25) | 2023 | USA | Qualitative study with thematic approach | 15 health care professionals (12 primary care physicians and 3 psychologists) | All type of mental disorders | - Normalizing discussion of mental health - Mental health care–seeking action - Using patient-centered Communication - Empathetic communication strategies - Sharing by health care professionals of their own experiences - Tailoring the discussion of mental health to patients’ preferred understanding - Build Trust and Rapport |

1. Raingruber B. Client and provider perspectives regarding the stigma of and nonstigmatizing interventions for depression. Archives of psychiatric nursing. 2002;16(5):201-7.

2. Angermeyer MC, Schulze B, Dietrich S. Courtesy stigma - A focus group study of relatives of schizophrenia patients. Social psychiatry and psychiatric epidemiology. 2003;38(10):593-602.

3. Caltaux D. Internalized stigma: A barrier to employment for people with mental illness. International Journal of Therapy and Rehabilitation. 2003;10(12):539-42.

4. Alvidrez J, Snowden LR, Kaiser DM. The experience of stigma among black mental health consumers. Journal of health care for the poor and underserved. 2008;19(3):874-93.

5. Kapungwe A, Cooper S, Mwanza J, Mwape L, Sikwese A, Kakuma R, et al. Mental illness--stigma and discrimination in Zambia. African journal of psychiatry. 2010;13(3):192-203.

6. Saillard EK. Psychiatrist views on stigmatization toward people with mental illness and recommendations. Turk Psikiyatri Dergisi. 2010;21(1):1-10.

7. Corbière M, Samson E, Villotti P, Pelletier JF. Strategies to fight stigma toward people with mental disorders: perspectives from different stakeholders. TheScientificWorldJournal. 2012;2012:516358.

8. Tawiah PE, Adongo PB, Aikins M. Mental Health-Related Stigma and Discrimination in Ghana: Experience of Patients and Their Caregivers. Ghana medical journal. 2015;49(1):30-6.

9. Krupchanka D, Kruk N, Murray J, Davey S, Bezborodovs N, Winkler P, et al. Experience of stigma in private life of relatives of people diagnosed with schizophrenia in the Republic of Belarus. Social psychiatry and psychiatric epidemiology. 2016;51(5):757-65.

10. Taghva A, Farsi Z, Javanmard Y, Atashi A, Hajebi A, Noorbala AA. Strategies to reduce the stigma toward people with mental disorders in Iran: Stakeholders' perspectives. BMC psychiatry. 2017;17(1).

11. Vedana KGG, Silva DRA, Miasso AI, Zanetti ACG, Borges TL. The Meaning of Stigma for People with Mental Disorders in Brazil. Issues in mental health nursing. 2017;38(12):1022-9.

12. Huggett C, Birtel MD, Awenat YF, Fleming P, Wilkes S, Williams S, et al. A qualitative study: experiences of stigma by people with mental health problems. Psychology and Psychotherapy: Theory, Research and Practice. 2018;91(3):380-97.

13. Bonsu AS, Salifu Yendork J. Community-Based Mental Health Care: Stigma and Coping Strategies Among Professionals and Family Caregivers in the Eastern Region of Ghana. Issues in mental health nursing. 2019;40(5):444-51.

14. Farsi Z, Taghva A, Butler SC, Tabesh H, Javanmard Y, Atashi A. Stigmatization toward patients with mental health diagnoses: Tehran's stakeholders' perspectives. Iranian journal of psychiatry and behavioral sciences. 2020;14(3).

15. Ong WJ, Shahwan S, Goh CMJ, Tan GTH, Chong SA, Subramaniam M. Daily Encounters of Mental Illness Stigma and Individual Strategies to Reduce Stigma - Perspectives of People With Mental Illness. Frontiers in psychology. 2020;11:590844.

16. Koschorke M, Oexle N, Ouali U, Cherian AV, Deepika V, Mendon GB, et al. Perspectives of healthcare providers, service users, and family members about mental illness stigma in primary care settings: A multisite qualitative study of seven countries in Africa, Asia, and Europe. PloS one. 2021;16(10 October).

17. Shahwan S, Tan GTH, Goh J, Subramaniam M. Strategies to Reduce Mental Illness Stigma-Perspectives of Individuals with Lived Experience. International Journal of Qualitative Methods. 2021;20:18-9.

18. Commey IT, Ninnoni JPK, Ampofo EA. Coping with personal care and stigma: experiences of persons living with schizophrenia. BMC nursing. 2022;21(1):107.

19. Lagunes-Cordoba E, Lagunes-Cordoba R, Fresan-Orellana A, Gonzalez-Olvera J, Jarrett M, Thornicroft G, et al. Mexican Psychiatric Trainees’ Attitudes Towards People with Mental Illness: A Qualitative Study. Community mental health journal. 2022;58(5):982-91.

20. Withers M, Jahangir T, Kubasova K, Ran MS. Reducing stigma associated with mental health problems among university students in the Asia-Pacific: A video content analysis of student-driven proposals. The International journal of social psychiatry. 2022;68(4):827-35.

21. Aiyub A, Jannah SR, Marthoenis M, Abdullah A, Sofyan H. Peer stigma, consequences, and anti-stigma strategies in adolescents with mental disorders: a qualitative investigation. Journal of Public Mental Health. 2023;22(2):60-72.

22. Arboleya-Faedo T, González-Menéndez A, González-Pando D, Paino M, Alonso-Pérez F. Experiences of Self-Stigma in People with Chronic Psychosis: A Qualitative Study. International journal of environmental research and public health. 2023;20(9).

23. Latifian M, Abdi K, Raheb G, Islam SMS, Alikhani R. The experiences of bipolar patients’ families regarding stigma coping strategies in Tehran: a qualitative study. Current Psychology. 2023.

24. Pederson AB, Fokuo JK, Thornicroft G, Bamgbose O, Ogunnubi OP, Ogunsola K, et al. Perspectives of university health care students on mental health stigma in Nigeria: Qualitative analysis. Transcultural psychiatry. 2023;60(2):272-85.

25. Phelan SM, Salinas M, Pankey T, Cummings G, Allen JP, Waniger A, et al. Patient and Health Care Professional Perspectives on Stigma in Integrated Behavioral Health: Barriers and Recommendations. Annals of family medicine. 2023;21(Suppl 2):S56-s60.
